# Use of Caffeine-Containing Energy Drinks by Japanese Middle School Students: A Cross-Sectional Study of Related Factors

**DOI:** 10.3390/nu15051275

**Published:** 2023-03-03

**Authors:** Satoko Yamasaki, Hiromi Kawasaki, Zhengai Cui

**Affiliations:** Graduate School of Biomedical and Health Sciences, Hiroshima University, Hiroshima 734-8551, Japan

**Keywords:** caffeine, energy drinks, Japan, middle school students, nutrition, sex differences, sleep

## Abstract

Excessive consumption of caffeine negatively affects individuals’ health. Therefore, we studied the use of energy drinks and the conditions associated with it among Japanese secondary school students. Participants were 236 students in grades 7–9 who completed anonymous questionnaires at home in July 2018. We measured the basic attributes and dietary, sleeping, and exercise habits. We used Chi-squared tests to compare differences between users and non-users of energy drinks. Logistic regression analyses were used to elucidate the complex association between the variables. The results showed that boys were more willing to consume energy drinks than girls. The reasons were ‘feeling fatigued’, ‘needing to stay awake’, ‘for curiosity’, and ‘to quench one’s thirst’. Among boys, the following were associated with the use of EDs. Buying their own snacks , not understanding nutritional labels on foods, high caffeinated beverage intake, always waking up at about the same time, and weight. Health guidance is needed to prevent overconsumption and dependence on energy drinks. The cooperation of parents and teachers is needed to achieve these goals.

## 1. Introduction

In recent years, several new beverages containing caffeine, called “energy drinks” (EDs), have come on the market [1]. In general, the constituent components of EDs are mainly caffeine, while other ingredients are isolation and combinations that include taurine, B complex vitamins, guarana, L-carnitine, and ginseng [2]. Caffeine can produce subjective reinforcement and discriminant stimuli similar to those produced by cocaine and amphetamines via dopamine [3]. After ingestion, caffeine is rapidly absorbed by the body and reaches a peak in plasma concentrations within 30 min of ingestion [4]. Excessive consumption of caffeine negatively affects individuals’ health, including caffeine intoxication, arrythmia and hypertension, and sleep disorders [5,6,7,8,9,10]. Caffeine levels of 400 mg or more for adults and 100–400 mg for children can cause anxiety, nausea, irritability, and increased nervousness [11,12]. As children are lighter in body weight and metabolize caffeine quicker than do adults, they are more susceptible to caffeine intoxication [13].

Previous studies have reported that people who drink caffeine have worse sleep quality, longer hours, and higher levels of stress than people who do not drink caffeine [14]. In an investigation of U.S. high school students, the use of EDs was related to attention deficit/hyperactivity disorder [15]. Other research revealed a correlation between juvenile caffeine consumption and aggressive behavior and behavioral disorders [16]. In an investigation of Spanish high school students, a worse school performance were risk factor for using EDs. [17]. In a study of New Zealand high school students, EDs consumption was significantly related to increased depressive symptoms [18,19]. There have also been reports that use of EDs by minors is related to alcohol, tobacco, and illegal drug use [20,21,22,23]. Thus, some fear that the use of EDs may be a gateway to using illegal drugs later on [24].

In Japan, there has been an increase in the number of emergency patients that had consumed massive amounts of caffeine-containing supplements and EDs [25]. In Japan, EDs have been sold as “soft drinks” since 2005, and the market is expanding annually. Convenience stores and vending machines allow both adults and children to purchase EDs without restrictions. Although studies outside of Japan have elucidated the adverse effects of overconsuming EDs, in Japan, there have been almost no studies on the harm of EDs, despite their consumption by Japanese children [26]. To avoid the negative effects of overusing EDs, it is important to understand the spread of EDs use, usage patterns, and the backgrounds of people who use them. The purpose of this study was to elucidate the use of EDs by Japanese children and the conditions related to their use. Although elementary school children tend to have their parents buy food and drinks for them, middle school students tend to buy their own food and drinks; therefore, we examined middle school students.

## 2. Materials and Methods

### 2.1. Study Population

This study was approved by the ethics committee of Hiroshima University (E-1118) and was conducted in accordance with the principles of the Declaration of Helsinki. Participants were middle school students (grades 7–9) from Prefecture A. Since they were minors, we obtained written consent from the students, their parents, and the school principal. Participants completed self-reported anonymous questionnaires at home. The study was conducted in July 2018.

We distributed 368 copies of the questionnaire forms and recovered 275 of them. The number of effective responses was 236. The final analysis thus included 236 students (126 girls): 80 grade-seven students (45 girls), 75 grade-eight students (41 girls), and 81 grade-nine students (40 girls) (Table 1).

### 2.2. Survey Contents

#### 2.2.1. Objective Variable

The objective variable was the use of EDs. Those who answered “have not used” EDs in the questionnaire form were regarded as the “non-user group”. Those who said they “have used” EDs comprised the “user group”.

#### 2.2.2. Explanatory Variables

The explanatory variables were basic attributes, lifestyle habits, and physical complaints. Items related to dietary habits included eating, sleeping, and exercise habits. For dietary habits, we used the Scale for the Evaluation of Healthy Eating Habits of Japanese People [27], consisting of four items about “dietary balance” and four items about “eating healthy”. Students evaluated themselves on a five-point scale (1–5). Total scores ranged from 12 to 60, with higher scores indicating healthier dietary behavior (Table 2).

As an additional indicator, we used participants’ “autonomic judgment” from the “Media Literacy Scale on Diet” [28]. The autonomic judgment subscales included the “use of food labeling” and “judgment of nutritional balance”. “Use of food labeling” consisted of the following four items: “choosing better food items by looking at nutritional content labels like calories etc.”, “choosing better food items by looking at the display of raw material expiration date”, “trying to look at food labels when buying food and drinks”, and “able to understand food labels”. “Judgment of nutritional balance” consisted of the following three items: “being careful about eating a balanced diet to remain healthy”, “trying to eat meals that consist of a staple food, a main dish, and side dishes”, and “able to judge whether your own dietary balance is good”. A five-point scale was used to evaluate these, and higher scores indicated higher media literacy.

To measure sleep, we used the Athens Insomnia Scale [29]. The questionnaire consists of eight items addressing “difficulty falling asleep”, “waking up in the middle of sleep”, “waking up early”, “total sleep time”, “quality of sleep”, “mood during the day”, “activities during the day”, and “drowsiness during the day”. Participants used a four-point scale to self-evaluate things that they experienced more than three times in the past month and assign a score from 0 to 3 for each item to evaluate sleep [30]. Higher total scores (range = 0–24) indicated worse sleep habits. Furthermore, past studies showed that behaviors that promote sleep included the following [31,32,33]: “always waking up at the same time [34]”, “getting sunlight after waking up in the morning [35]”, “not consuming caffeine before sleep [36,37,38]”, “not taking naps in the afternoon [39]”, “having an early bath [32]”, “not having a snack after dinner [40]”, “not looking at screens like smartphones, television, or videogames before sleep [32]”, and “not thinking about problems or unhappy events before sleeping [31,33]”. Therefore, we calculated a total score for behavior to promote sleep by considering what “they were doing every day” as three points, what “they were not doing much every day” as two points, and what “they were unable to do at all” as one point. Since sleeping time and waking time is based on a continuous scale, we switched to a decimal system.

### 2.3. Statistical Analysis

We examined the sex differences in the above variables. Chi-squared (χ^2^) tests were used for comparisons. Questions were answered on a Likert scale of 2 or 4. For example, applicable every day = 1 and other = 2. A five-point scale (from 1 “not at all applicable” to 5 “very applicable”) was used. These were also divided into “yes” and “no” answers. A logistic regression analysis was conducted to elucidate the complex association between variables. The objective variable was the use of EDs: “use” was one; “no use” was zero. As dependent variables, we selected variables that had a *p*-value < 0.20 on the χ^2^-test and Mann–Whitney U-test. For the independent variable in the logistic regression analysis, the forced input method was used; then, the variable increase method using the likelihood ratio test was used. Two-tailed significance was set at *p* < 0.05. Analyses were performed using SPSS version 25.0 (IBM).

## 3. Results

### 3.1. Student Overview

A summary of the characteristics of the students in this study is presented in Table 1.

**Table 1:** Student overview.

Item	Total	Boys	Girls
	236 (%)	110 (%)	126 (%)
Grade level			
Grade 7	80 (33.9)	35 (31.8)	45 (35.8)
Grade 8	75 (31.8)	34 (30.9)	41 (32.5)
Grade 9	81 (34.3)	41 (37.3)	40 (31.7)
Frequency of eating breakfast			
Not every day	26 (11.0)	12 (10.9)	14 (11.1)
Every day	210 (89.0)	98 (89.0)	112 (88.9)
Number of snacks eaten daily			
More than once	202 (85.6)	93 (84.5)	109 (86.5)
No snacking	34 (14.4)	17 (15.5)	17 (13.5)
Frequency of buying snacks and juice on one’s own			
Do not buy	79 (33.5)	35 (31.8)	44 (34.9)
More than once a week	157 (66.5)	75 (68.2)	82 (65.1)
Know the effects of caffeine			
Do not know/have heard of it	88 (37.3)	42 (38.2)	46 (36.5)
Know a little/know/can explain	148 (62.7)	68 (61.8)	80 (63.5)
Use smartphone in bed			
No	181 (76.7)	82 (74.5)	99 (78.6)
Yes	55 (23.3)	28 (25.5)	27 (21.4)
Haziness			
Often/sometimes	85 (36.0)	39 (35.5)	46 (36.5)
Never/almost never	151 (64.0)	71 (64.5)	80 (63.5)
Diarrhea			
Often/sometimes	54 (22.9)	30 (27.3)	24 (19.0)
Never/almost never	182 (77.1)	80 (72.7)	102 (81.0)
Dizziness			
Often/sometimes	94 (39.8)	41 (37.3)	53 (42.1)
Never/almost never	142 (60.2)	69 (62.7)	73 (57.9)
Jumpy eyelids or muscles			
Often/sometimes	73 (30.9)	28 (25.5)	45 (35.7)
Never/almost never	163 (69.1)	82 (74.5)	81 (64.3)
Fast heartbeat for no known reason			
Often/sometimes	27 (11.4)	14 (12.7)	13 (10.3)
Never/almost never	209 (88.6)	96 (87.3)	113 (89.7)
Sudden shortness of breath			
Often/sometimes	19 (8.1)	6 (5.5)	13 (10.3)
Never/almost never	217 (91.9)	104 (94.5)	113 (89.7)
Tremors and numbness of hands/feet			
Often/sometimes	35 (14.8)	16 (14.5)	19 (15.1)
Never/almost never	201 (85.2)	94 (85.5)	107 (84.9)
Understand food labels *			
Completely disagree/disagree/neither	63 (26.7)	35 (31.8)	28 (22.2)
Strongly agree/slightly agree	173 (73.3)	75 (68.2)	98 (77.8)
Try to look at food labels when buying food and beverages			
Completely disagree/disagree/neither	107 (45.3)	64 (58.2)	43 (34.1)
Strongly agree/slightly agree	129 (54.7)	46 (41.8)	83 (65.9)
Can select better food items by looking at the label ingredient name and expiration dates.			
Completely disagree/disagree/neither	75 (31.8)	48 (43.6)	27 (21.4)
Strongly agree/slightly agree	161 (68.2)	62 (56.4)	99 (78.6)
Can select better food items by looking at nutritional labels like calories, etc.			
Completely disagree/disagree/neither	117 (49.6)	64 (58.2)	53 (42.1)
Strongly agree/slightly agree	119 (50.4)	46 (41.8)	73 (57.9)
Try to eat meals containing staple foods, a main dish, and side dishes			
Completely disagree/disagree/neither	84 (35.6)	43 (39.1)	41 (32.5)
Strongly agree/slightly agree	152 (64.4)	67 (60.9)	85 (67.5)
Can judge if my diet is balanced			
Completely disagree/disagree/neither	79 (33.5)	39 (35.5)	40 (31.7)
Strongly agree/slightly agree	157 (66.5)	71 (64.5)	86 (68.3)
Do feel you want to get more exercise?			
Not applicable/slightly applicable	55 (23.3)	26 (23.6)	29 (23.0)
Somewhat applicable/applicable	181 (76.7)	84 (76.4)	97 (77.0)
Can you make time to exercise?			
Not applicable/slightly applicable	96 (40.7)	32 (29.1)	64 (50.8)
Somewhat applicable/applicable	140 (59.3)	78 (70.9)	62 (49.2)
Always able to wake up at the same time			
Not applicable/not very applicable	52 (22.0)	24 (21.8)	28 (22.2)
Applicable every day	184 (78.0)	86 (78.2)	98 (77.8)
Able to get sunlight after waking up			
Not applicable/not very applicable	157 (66.5)	64 (58.2)	93 (73.8)
Applicable every day	79 (33.5)	46 (41.8)	33 (26.2)
Do not consume caffeine four hours before sleep			
Not applicable/not very applicable	109 (46.2)	50 (45.5)	59 (46.8)
Applicable every day	127 (53.8)	60 (54.5)	67 (53.2)
Do not nap in the afternoons			
Not applicable/not very applicable	73 (30.9)	27 (24.5)	46 (36.5)
Applicable every day	163 (69.1)	83 (75.5)	80 (63.5)
Take early baths			
Not applicable/not very applicable	170 (72.0)	73 (66.4)	97 (77.0)
Applicable every day	66 (28.0)	37 (33.6)	29 (23.0)
Not snacking after dinner			
Not applicable/not very applicable	91 (38.6)	44 (40.0)	47 (37.3)
Applicable every day	145 (61.4)	66 (60.0)	79 (62.7)
Stop smartphone/TV/game/computer use one hour before sleep			
Not applicable/not very applicable	176 (74.6)	87 (79.1)	89 (70.6)
Applicable every day	60 (25.4)	23 (20.9)	37 (29.4)
Not thinking worries and unhappy events before sleep			
Not applicable/not very applicable	129 (54.7)	59 (53.6)	70 (55.6)
Applicable every day	107 (45.3)	51 (46.4)	56 (44.4)
Are there any current lifestyle habits that you could improve upon?			
No	144 (61.0)	58 (52.7)	86 (68.3)
Yes	92 (39.0)	52 (47.3)	40 (31.7)

* Understand food labels: raw ingredient names, expiry dates, nutritional labels, etc.

### 3.2. Concerning Using EDs

EDs were used by 53 boys (48.2%) and 34 girls (27.0%). χ^2^-tests revealed that male students were using EDs at a significantly greater rate than female students (*p* = 0.001). Thus, we analyzed the data by sex. Furthermore, the use of EDs by sex was non-significantly different between grade levels (overall, *p* = 0.217; boys, *p* = 0.232; girls, *p* = 0.849).

Concerning frequency among those who were using EDs, 5.6% of boys and 0% of girls used them every day, 5.6% of boys and 5.9% of girls used them once or more per week, and 88.9% of boys and 94.1% of girls used them one to three times per month. The response rate to the frequency question was not good. The percentage was calculated only for the students who responded. Concerning when they consumed EDs, 18.9% of boys and 17.6% of girls stated, “when I am tired”, 15.1% of boys and 11.8% of girls stated, “when I am sleepy/when I have to stay awake”, 11.3% of boys and 5.9% of girls stated “when I am thirsty”, 9.4% of boys and 8.8% of girls stated “out of curiosity”, 3.8% of boys and 2.9% of girls stated “when I am feeling sick”, 3.8% of boys and 2.9% of girls stated “when I cannot feel motivated”, 3.8% of boys and 11.8% of girls stated “after exercise”, 3.8% of boys and 11.8% of girls stated “before tests/to study”, 1.9% of boys and 5.9% of girls stated “when I am given one”, 3.8% of boys and 2.9% of girls stated “when I want to drink one”, and 7.5% of boys and 0% of girls had “other” reasons (17.0% of boys and 17.6% of girls did not answer). The one student that used EDs every day said that he consumed six EDs each time he used them.

### 3.3. Relationship between EDs Use and Lifestyle in Nominal Scale

Table 2 shows the relationship between EDs use and lifestyle habits (nominal scale). Boys with use had significantly higher percentages than those without use for buying their own snacks and juice (*p* = 0.001), not understanding food labels (*p* = 0.035), and always getting up at about the same time (*p* = 0.035). No significantly related items due to use were found for the female students. Among boys, the user group had a significantly higher proportion of students that bought snacks and juice on their own, did not understand nutritional content, and woke up at almost the same time every day than did the non-user group. There were no group differences among the female students.

**Table 2:** Relationship between EDs use and lifestyle habits in nominal scale.

Item	Total *n*	Boys			Girls		
		Non- Users *n* (%)	Users *n* (%)	*p*	Non- Users *n* (%)	Users *n* (%)	*p*
Grade level							
Grade 7	80	21 (36.8)	14 (26.4)	0.232	33 (35.9)	12 (35.3)	0.849
Grade 8	75	19 (33.3)	15 (28.3)		31 (33.7)	10 (29.4)	
Grade 9	81	17 (29.8)	24 (45.3)		28 (30.4)	12 (35.3)	
Frequency of eating breakfast							
Not every day	26	6 (10.5)	6 (11.3)	0.894	9 (9.8)	5 (14.7)	0.435
Every day	210	51 (89.5)	47 (88.7)		83 (90.2)	29 (85.3)	
Number of snacks eaten daily							
More than once	202	46 (80.7)	47 (88.7)	0.247	78 (84.8)	31 (91.2)	0.351
No snacking	34	11 (19.3)	6 (11.3)		14 (15.2)	3 (8.8)	
Frequency of buying snacks and juice on one’s own							
Do not buy	79	26 (45.6)	9 (17.0)	0.001	35 (38.0)	9 (26.5)	0.226
More than once a week	157	31 (54.4)	44 (83.0)		57 (62.0)	25 (73.5)	
Know the effects of caffeine							
Do not know/have heard of it	88	18 (31.6)	24 (45.3)	0.139	34 (37.0)	12 (35.3)	0.863
Know a little/know/can explain	148	39 (68.4)	29 (54.7)		58 (63.0)	22 (64.7)	
Use smartphone in bed							
No	181	46 (80.7)	36 (67.9)	0.124	70 (76.1)	29 (85.3)	0.264
Yes	55	11 (19.3)	17 (32.1)		22 (23.9)	5 (14.7)	
Haziness							
Often/sometimes	85	20 (35.1)	19 (35.8)	0.934	32 (34.8)	14 (41.2)	0.508
Never/almost never	151	37 (64.9)	34 (64.2)		60 (65.2)	20 (58.8)	
Diarrhea							
Often/sometimes	54	18 (31.6)	12 (22.6)	0.293	17 (18.5)	7 (20.6)	0.789
Never/almost never	182	39 (68.4)	41 (77.4)		75 (81.5)	27 (79.4)	
Dizziness							
Often/sometimes	94	22 (38.6)	19 (35.8)	0.766	40 (43.5)	13 (38.2)	0.597
Never/almost never	142	35 (61.4)	34 (64.2)		52 (56.5)	21 (61.8)	
Jumpy eyelids or muscles							
Often/sometimes	73	13 (22.8)	15 (28.3)	0.509	34 (37.0)	11 (32.4)	0.632
Never/almost never	163	44 (77.2)	38 (71.7)		58 (63.0)	23 (67.6)	
Fast heartbeat for no known reason							
Often/sometimes	27	9 (15.8)	5 (9.4)	0.318	8 (8.7)	5 (14.7)	0.325
Never/almost never	209	48 (84.2)	48 (90.6)		84 (91.3)	29 (85.3)	
Sudden shortness of breath							
Often/sometimes	19	3 (5.3)	3 (5.7)	0.927	8 (8.7)	5 (14.7)	0.325
Never/almost never	217	54 (94.7)	50 (94.3)		84 (91.3)	29 (85.3)	
Tremors and numbness of hands/feet							
Often/sometimes	35	8 (14.0)	8 (15.1)	0.875	16 (17.4)	3 (8.8)	0.233
Never/almost never	201	49 (86.0)	45 (84.9)		76 (82.6)	31 (91.2)	
Understand food labels *							
Completely disagree/disagree/neither	63	13 (22.8)	22 (41.5)	0.035	20 (21.7)	8 (23.5)	0.830
Strongly agree/slightly agree	173	44 (77.2)	31 (58.5)		72 (78.3)	26 (76.5)	
Try to look at food labels when buying food and beverages							
Completely disagree/disagree/neither	107	32 (56.1)	32 (60.4)	0.653	32 (34.8)	11 (32.4)	0.798
Strongly agree/slightly agree	129	25 (43.9)	21 (39.6)		60 (65.2)	23 (67.6)	
Can select better food items by looking at the label ingredient name and expiration dates							
Completely disagree/disagree/neither	75	22 (38.6)	26 (49.1)	0.269	20 (21.7)	7 (20.6)	0.889
Strongly agree/slightly agree	161	35 (61.4)	27 (50.9)		72 (78.3)	27 (79.4)	
Can select better food items by looking at nutritional labels like calories, etc.							
Completely disagree/disagree/neither	117	31 (54.4)	33 (62.3)	0.403	37 (40.2)	16 (47.1)	0.490
Strongly agree/slightly agree	119	26 (45.6)	20 (37.7)		55 (59.8)	18 (52.9)	
Try to eat meals containing staple foods, a main dish, and side dishes							
Completely disagree/disagree/neither	84	23 (40.4)	20 (37.7)	0.779	31 (33.7)	10 (29.4)	0.649
Strongly agree/slightly agree	152	34 (59.6)	33 (62.3)		61 (66.3)	24 (70.6)	
Can judge if my diet is balanced							
Completely disagree/disagree/neither	79	20 (35.1)	19 (35.8)	0.934	32 (34.8)	8 (23.5)	0.228
Strongly agree/slightly agree	157	37 (64.9)	34 (64.2)		60 (65.2)	26 (76.5)	
Do feel you want to get more exercise?							
Not applicable/slightly applicable	55	14 (24.6)	12 (22.6)	0.813	20 (21.7)	9 (26.5)	0.575
Somewhat applicable/applicable	181	43 (75.4)	41 (77.4)		72 (78.3)	25 (73.5)	
Can you make time to exercise?							
Not applicable/slightly applicable	96	16 (28.1)	16 (30.2)	0.807	47 (51.1)	17 (50.0)	0.914
Somewhat applicable/applicable	140	41 (71.9)	37 (69.8)		45 (48.9)	17 (50.0)	
Always able to wake up at the same time							
Not applicable/not very applicable	52	17 (29.8)	7 (13.2)	0.035	17 (18.5)	11 (32.4)	0.096
Applicable every day	184	40 (70.2)	46 (86.8)		75 (81.5)	23 (67.6)	
Able to get sunlight after waking up							
Not applicable/not very applicable	157	34 (59.6)	30 (56.6)	0.746	65 (70.7)	28 (82.4)	0.185
Applicable every day	79	23 (40.4)	23 (43.4)		27 (29.3)	6 (17.6)	
Do not consume caffeine four hours before sleep							
Not applicable/not very applicable	109	22 (38.6)	28 (52.8)	0.134	41 (44.6)	18 (52.9)	0.403
Applicable every day	127	35 (61.4)	25 (47.2)		51 (55.4)	16 (47.1)	
Do not nap in the afternoons							
Not applicable/not very applicable	73	12 (21.1)	15 (28.3)	0.377	33 (35.9)	13 (38.2)	0.807
Applicable every day	163	45 (78.9)	38 (71.7)		59 (64.1)	21 (61.8)	
Take early baths							
Not applicable/not very applicable	170	38 (66.7)	35 (66.0)	0.944	69 (75.0)	28 (82.4)	0.384
Applicable every day	66	19 (33.3)	18 (34.0)		23 (25.0)	6 (17.6)	
Not snacking after dinner							
Not applicable/not very applicable	91	27 (47.4)	17 (32.1)	0.102	32 (34.8)	15 (44.1)	0.336
Applicable every day	145	30 (52.6)	36 (67.9)		60 (65.2)	19 (55.9)	
Stop smartphone/TV/game/computer use one hour before sleep							
Not applicable/not very applicable	176	42 (73.7)	45 (84.9)	0.148	65 (70.7)	24 (70.6)	0.994
Applicable every day	60	15 (26.3)	8 (15.1)		27 (29.3)	10 (29.4)	
Not thinking worries and unhappy events before sleep							
Not applicable/not very applicable	129	31 (54.4)	28 (52.8)	0.870	48 (52.2)	22 (64.7)	0.209
Applicable every day	107	26 (45.6)	25 (47.2)		44 (47.8)	12 (35.3)	
Are there any current lifestyle habits that you could improve upon?							
No	144	33 (57.9)	25 (47.2)	0.260	61 (66.3)	25 (73.5)	0.439
Yes	92	24 (42.1)	28 (52.8)		31 (33.7)	9 (26.5)	

Number and proportion of people (%) are shown for each question. χ^2^-tests were used for analyses between the energy drink user/non-user groups. * Understand food labels: raw ingredient names, expiry dates, nutritional labels, etc.

### 3.4. Relationship between ED Use and Lifestyle on a Continuous Scale

Table 3 shows the relationship between use of EDs and lifestyle habits (continuous scale). The with-use group was significantly heavier (*p* = 0.031), using more caffeinated beverages such as coffee (*p* = 0.010), and waking up later on holidays (*p* = 0.031) than the without-use group. Among the female students, “dietary balance”, a sub-item of the Japanese Healthy Eating Behavior Rating Scale, was significantly higher in the group with use than in the group without use.

Among boys, the user group had significantly heavier body weight, consumed more caffeine-containing drinks, and woke up later on holidays than the non-user group. Among girls, dietary balance—a sub-item of the Scale for the Evaluation of Healthy Eating Habits of Japanese People—was significantly higher in the user group than in the non-user group.

### 3.5. The Results of the Logistic Regression Analysis concerning the Factors Related to Energy Drink Use among Boys and Girls

Table 4 show the results of the logistic regression analysis concerning the factors related to energy drink use among boys and girls. Based on the χ^2^ test and Mann–Whitney results,” Wake up almost at the same time every day”, “Buy juice and snacks on your own”, “Able to read food labels”, and “Bedtime on weekdays (continuous data) were selected for boys. For girls, “Wakes up almost at the same time every day”, “Able to get sunlight after waking up”, “Scale for the Evaluation of Healthy Eating Habits of Japanese People (dietary balance)”, “Media Literacy Scale on Diet (judgment of nutritional balance)”, “Number of cups of caffeine”, and “Amount of sleep on holidays (continuous data)” were selected. Among the male students, the odds ratio for EDs use was 6.34 (95% CI: 1.86–21.67) (*p* = 0.003) for students who reported waking up at the same time every day. The odds ratio for EDs use was 4.26 (95% CI: 1.63–11.15) (*p* = 0.003) for students who reported buying their own juice or snacks at least once a week. The odds ratio for having EDs use was 0.29 (95% CI: 0.11–0.80) (*p* = 0.017) for students who reported being able to understand food labels. The odds ratio for students of late bedtime on school nights was 1.69 (95% CI: 0.99–2.87) (*p* = 0.054).

In contrast, the odds ratio for EDs use among female students who reported waking up at the same time each day was 0.36 (95% CI: 0.13–0.95) (*p* = 0.039).

Compared to their counterparts, the odds ratios of boys that used EDs were greater for those that woke up at the same time every day, bought snacks and juice by themselves more than once a week, understood food labels. Aside from “Wakes up almost at the same time every day,” no significant differences were found concerning female students. 

## 4. Discussion

A study of EDs use and lifestyle habits among middle school students found that EDs use was significantly higher among boys. Among boys, the following were associated with the use of EDs. Buying their own snacks , not understanding nutritional labels on foods, high caffeinated beverage intake, late bedtimes on weekdays, always waking up at about the same time, and weight. Among girls, students with a well-balanced diet were significantly more likely to be EDs users.

There was significantly greater EDs use among male students than among female students. Similar results have been reported in studies in the U.S. and Canada in adolescent students [20,21,22,41,42]. It has been reported that boys are more likely to feel the effects of caffeine than girls and that the effects of caffeine are more likely to appear in boys [43], which led to the assumption that boys were the target for purchasing the product, which was one of the reasons why boys were more likely to use EDs [44].

EDs are also widely marketed to promote energy as well as mental and physical endurance and performance. EDs can be classified as dietary supplements, and about 1/3rd of 12–17-year-olds (teens) used EDs [45,46]. In addition, according to the analysis of this study, female junior high school students are more than twice as likely as boys to be aware of their body shape and obesity [47], which suggests that they pay attention to the nutritional balance of their meals and are more regular in their diet. On the other hand, boys often purchase snacks, soft drinks, and sweet breads in addition to meals [48]. This was also assumed to be the cause.

Middle school students are in the period when the foundations of desirable lifestyle habits such as exercise, diet, and sleep are being formed. The acquisition of good lifestyle habits by junior high school students is one of the major challenges in school health. However, in recent years, there have been indications of deterioration in the sleeping conditions of children, including delayed sleep onset and decreased sleep duration [49]. Prior research has shown that poor sleep status is known to be a risk factor for the development of underlying and lifestyle-related diseases, and may lead to lower learning performance and self-esteem in junior high school students [50,51]. In addition, caffeine intake status may play a role in the deterioration of sleep conditions. Studies of children overseas have shown that those with high daily caffeine intake report higher rates of difficulty falling asleep, difficulty waking up, daytime sleepiness, and other poor sleep conditions [6,52]. The results also suggested that EDs use is related to waking up late on holidays (i.e., non-school days). This lateness is presumed to be a way to compensate for the lack of sleep on a daily basis by sleeping longer on holidays. Furthermore, the results suggest that students are using EDs to suppress drowsiness or to improve academic performance. Given that academic performance is negatively correlated with higher EDs consumption [53], students should be encouraged to go to sleep earlier and wake up earlier to reduce drowsiness rather than using EDs to force themselves to stay awake.

However, going to bed early and getting up early is one of the most difficult tasks for junior high school students. Students need to be motivated to live autonomously and balance their studies and hobbies. Attachment to family increases autonomy, while life management by preference decreases autonomy [54]. One of the concepts of autonomy includes self-control. Since the higher the self-control, the higher the sense of well-being [55], control of one’s life by non-self-motivated materials should be avoided. The high prevalence of EDs use among middle school students who buy their own snacks also requires support from families to ensure that middle school boys in particular do not leave their parents’ guidance and supervision too quickly. Even though the EDs users in this study went to bed late, they woke up at the same time in the morning, inferring that they received adequate family support.

In recent years, there has been concern about the health problems associated with excessive caffeine intake in children. Caffeine intake guidelines have been established in Western countries [56,57], tolerable doses have been indicated, and advertising and marketing rules have been tightened for caffeine-rich beverages and supplements. On the other hand, in Japan, although there are no guidelines regarding the amount of caffeine consumed, the Ministry of Health, Labor and Welfare and the Ministry of Agriculture, Forestry and Fisheries have issued warnings regarding excessive caffeine intake, and have recommended that beverages with caffeine concentrations above a certain level be labeled to prevent excessive intake [58]. In recent years, in Japan, 60–70% of the individuals arrested for using tablet-form synthetic drugs such as cannabis, methylenedioxymethamphetamine (MDMA), etc. have been minors or young people in their 20s [59]. Cannabis, MDMA, and other drugs are abused mainly by young people, and some fear that the use of EDs may be a gateway to future illegal drug use [24]. Consequently, students should be educated about the impacts that overconsumption of EDs and caffeine can have so they can exercise caution if they choose to consume EDs. Labeling adopted in other countries should be recognized as necessary in Japan.

Prior research has shown that SNS was a factor influencing the purchasing behavior of young people [60]. It has also been reported that those who have experienced EDs intake have a higher rate of SNS use than those who have not experienced EDs intake [61]. Therefore, we also believe that a certain level of social regulation regarding EDs-related advertising and sales is necessary.

EDs use was related to high consumption of other caffeine-containing drinks, indicating a risk of caffeine overconsumption. Health guidance should be provided to these individuals to prevent overconsumption and dependence. Since students’ ability to buy food by themselves was related to EDs consumption, students should receive guidance concerning their purchases at a young age (i.e., when in elementary school) to promote healthier decision-making. In addition, significantly more boys do not understand food levels, the use of EDs was related to not understanding the nutritional labels. Students need to be taught about caffeine levels and the effects that caffeine has on their bodies. Parents and physical education teachers should collaborate to ensure children understand the ingredients and nutritional value of what they are consuming. In contrast, among girls, EDs use was associated with better dietary balance. The girls indicate that those interested in the diet understand and use the ingredients. Self-medication plays an important role in controlling life for girls, including countermeasures for menstrual cramps and premenstrual tension [48,62]. We suggest the possibility of learning objectively about interest in caffeine as well.

This study had some limitations. First, this study is a cross-sectional study, and thus, causal relationships between variables cannot be clarified. Second, we employed a small sample size, and only one school was analyzed; thus, selection bias is a possibility. Furthermore, the EDs user group included those who consumed EDs regularly or occasionally. Going forward, it is necessary to increase the sample size, to examine middle school students and high school students, and elucidate the characteristics of regular users to advance research that helps prevent health damages due to the overconsumption of EDs.

## 5. Conclusions

In this exploratory study, we examined factors associated with EDs use among Japanese middle school students. The use of EDs was significantly greater among boys than girls. Among boys, the use of EDs was related to waking up at the same time every day, buying snacks and drinks by themselves, and not understanding food labels. It was suggested that students who forcibly suppress their drowsiness are exposed to the risk of drinking EDs.

## Figures and Tables

**Table 3:** Relationship between EDs use and lifestyle habits on a continuous scale.

Item	Boys			Girls		
	Non-User M * (SD **)	User M * (SD **)	*p* ***	Non-User M * (SD **)	User M * (SD **)	*p* ***
Height	159.01 (7.32)	161.84 (7.13)	0.055	154.35 (5.01)	154.26 (5.09)	0.739
Body weight	47.46 (9.90)	50.281 (9.18)	0.031	44.61 (6.79)	44.89 (6.93)	0.498
Body mass index	18.70 (3.32)	19.09 (2.65)	0.140	18.68 (2.32)	18.76 (2.16)	0.690
Media-viewing time (min.)						
on school days	92.63 (61.49)	92.45 (56.63)	0.896	76.52 (51.99)	75.15 (46.75)	0.916
on holidays	175.96 (95.29)	182.26 (120.28)	0.966	137.28 (89.79)	136.76 (78.27)	0.692
Exercise time (min.)	111.23 (58.68)	120.85 (64.46)	0.392	77.64 (53.95)	70.88 (60.72)	0.310
Number of consumed cups of drinks containing caffeine (200 mL)	1.14 (1.47)	1.85 (1.63)	0.010	1.39 (1.67)	1.32 (1.61)	0.943
Scale for the Evaluation of Healthy Eating Habits of Japanese People						
dietary balance	16.35 (3.30)	16.23 (3.43)	0.894	16.33 (3.49)	17.62 (2.86)	0.049
eating healthy meals	14.47 (3.07)	13.57 (3.04)	0.101	14.97 (3.28)	15.71 (2.55)	0.320
food restrictions for health	10.21 (3.52)	10.42 (3.58)	0.658	11.0 (3.59)	11.24 (3.71)	0.832
total	41.04 (7.78)	40.21 (7.33)	0.518	42.29 (8.24)	44.56 (7.64)	0.161
Media Literacy Scale on Diet						
use of food labels	16.61 (2.08)	16.25 (2.20)	0.328	16.67 (1.94)	16.74 (2.09)	0.978
judgment of nutritional balance	12.00 (2.04)	12.26 (2.13)	0.502	12.17 (2.00)	12.79 (1.67)	0.135
total of autonomic judgment	28.61 (3.29)	28.51 (3.79)	0.874	28.85 (3.35)	29.53 (2.99)	0.239
Amount of sleep (min.)						
on school days	442.63 (54.49)	440.09 (60.00)	0.882	417.75 (53.23)	415.59 (50.09)	0.905
on holidays	521.32 (81.71)	534.62 (75.24)	0.314	513.15 (60.49)	497.50 (67.67)	0.068
Wake time						
on school days	6.24 (0.81)	6.49 (0.62)	0.059	6.22 (0.70)	6.33 (0.73)	0.416
on holidays	7.72 (1.28)	8.24 (1.29)	0.031	7.72 (0.99)	7.62 (1.11)	0.475
Bedtime						
on school nights	10.87 (0.83)	11.15 (0.87)	0.053	11.25 (0.81)	11.40 (0.85)	0.663
on holidays	11.04 (0.91)	11.33 (0.90)	0.110	11.16 (0.99)	11.32 (1.07)	0.508
Athens insomnia scale	11.89 (2.23)	11.60 (2.19)	0.537	11.53 (2.47)	12.18 (2.80)	0.201
Behavior to promote sleep	18.67 (2.54)	18.47 (2.55)	0.729	18.21 (2.78)	17.65 (3.04)	0.462

* M: mean. ** SD: standard deviation. *** *p*: χ^2^-tests were used for analyses between the energy drink user/non-user groups.

**Table 4:** Factors related to energy drink use in boys and girls as per the logistic regression analysis results.

Item	Boys			Girls		
	OR *	95% CI **	*p* ***	OR *	95% CI **	*p* ****
Wakes up almost at the same time every day						
Not applicable/not very applicable	Ref.	----	----	Ref.	----	----
Applicable every day (1)	6.34	1.86–21.67	0.003	0.36	0.13–0.95	0.039
Buy juice and snacks on your own						
Do not buy	Ref.	----	----	----	----	----
Buy more than once a week (1)	4.26	1.63–11.15	0.003	----	----	----
Able to read food labels						
Completely disagree/disagree/neither	Ref.	----	----	----	----	----
Strongly agree/slightly agree (1)	0.294	0.11–0.80	0.017	----	----	----
Bedtime on school nights	1.688	0.99–2.87	0.054	----	----	----
Able to get sunlight after waking up						
Not applicable/not very applicable	----	----	----	Ref.	----	----
Applicable every day	----	----	----	0.38	0.13–1.10	0.075
Scale for the Evaluation of Healthy Eating Habits of Japanese People (dietary balance)	----	----	----	1.14	0.95–1.38	0.165
Media Literacy Scale on Diet (judgment of nutritional balance)	----	----	----	1.12	0.82–1.53	0.478
Number of cups of caffeine	----	----	----	1.05	0.81–1.37	0.704
Amount of sleep on holidays	----	----	----	1.00	0.99–1.00	0.152

* OR: odd radio. ** 95%CI: 95% confidence interval. *** *p*: The analysis used a stepwise method of increasing variables in logistic regression analysis (likelihood ratio). ***** *p*: Forced entry method of logistic regression analysis was used.

## Data Availability

The datasets used and/or analyzed during the current study are available from the corresponding author on reasonable request.
